# Continuous renal replacement therapy in a neonate with methylmalonic acidemia and severe hyperammonemia: A case report

**DOI:** 10.1097/MD.0000000000042046

**Published:** 2025-07-25

**Authors:** Bingchun Lin, Yichu Huang, Yanliang Yu, Xiaoyun Xiong, Chuanzhong Yang

**Affiliations:** a Department of Neonatology, Affiliated Shenzhen Maternity & Child Healthcare Hospital, Southern Medical University, Shenzhen, Guangdong Province, China.

**Keywords:** continuous renal replacement therapy, hemodialysis, hyperammonemia, methylmalonic acidemia, neonate

## Abstract

**Background:**

Methylmalonic acidemia (MMA) is a rare disease that is often misdiagnosed and overlooked. Metabolic acidosis and severe hyperammonemia are severe morbidities of MMA that demand immediate treatment.

**Case-diagnosis:**

Here, we describe an MMA neonate with severe hyperammonemia and acidosis that was successfully rescued by continuous renal replacement therapy. A 6-day-old male neonate, with a gestational age of 39 + 3 weeks and a birth weight of 2880 g, was admitted to the emergency with the symptoms of “cyanosis of the face and coma for 2 hours.” The infant was suffering from hypoglycemia, hypocalcemia, severe hyperammonemia, and severe metabolic acidosis. After admission, the blood ammonia continued to rise, and continuous renal replacement therapy was promptly administered until the patient gradually regained consciousness. Blood tandem mass spectrometry and profiles revealed that methylmalonic acid and methyl citrate levels were significantly enhanced. Two heterozygous mutations were detected in the MUT gene, confirming the diagnosis of methylmalonic acidemia mut.

**Conclusions:**

The patient’s symptoms gradually improved after receiving vitamin B12, l-carnitine, and specialized milk feeding. The patient was successfully discharged from the hospital after 41 days.

## 1. Introduction

Due to the low incidence and absence of specificity in the clinical symptoms, methylmalonic acidemia (MMA) is prone to misdiagnosis and being overlooked. MMA can lead to acute life-threatening consequences such as severe hyperammonemia and metabolic acidosis, necessitating immediate medical treatment. Here, we describe an MMA neonate with severe hyperammonemia and acidosis that was successfully rescued by continuous renal replacement therapy (CRRT).

## 2. Case presentation

A 6-day-old male neonate with a gestational age of 39 + 3 weeks and a birth weight of 2880 g was admitted to the emergency department due to the symptoms of “face-colored cyanosis with 2 hours of coma.” The vital signs were recorded (body temperature 34 °C, heart rate 120 bpm, respiratory rate 54, blood pressure 45/30 mm Hg). The pupil was 2.5 mm in diameter and of a large circular shape with a light reflex. The emergency examination revealed that the peripheral blood glucose level was 1.6 mmol/L, the blood ammonia level was 1168 μmol/L, the blood calcium level was 0.63 mmol/L, the blood gas analysis pH was 7.26, the alkali reserve was −17.5 mmol/L, and the anion gap was 35.2 mmol/L, indicating that the infant was suffering from hypoglycemia, hypocalcemia, severe hyperammonemia, and severe metabolic acidosis. Despite treatment with fluid infusion and arginine supplements, the patient’s blood ammonia continued to rise to 2340.2 μmol/L.

CRRT was initiated at 12 hours after admission. A continuous arteriovenous hemodialysis mode was applied, and catheterization was carried out via peripheral arteriovenous access. After being prefilled with normal saline, the pipeline and filter were prefilled with erythrocyte and plasma (2:1, 75 mL). The blood pump speed was 10 mL/min, while the dialysis pump was 100 mL/h. Sodium citrate was used for anticoagulation. CRRT treatment lasted for 24 hours, with blood gas analysis monitored every 2 hours, and blood ammonia, blood routine, coagulation function, electrolyte, and renal function indicators measured every 4–6 hours (Table 1).

**Table 1:** Changes of various results before, during and after CRRT treatment.

	Before CRRT	CRRT 6 h	CRRT 12h	CRRT 18 h	CRRT end
Blood pressure (mm Hg)	56/28 (37)	46/36 (39)	74/42 (54)	66/38 (50)	69/40 (53)
Alkali residue (mmol/L)	‐19.7	‐11	‐13.6	‐12.4	‐8.0
Lactic acid (mmol/L)	1.7	2.2	2.8	5.4	4.7
Urea (mmol/L)	10.3	–	2.16	0.95	0.71
Creatinine (μmol/L)	112	–	27	22	24
Blood ammonia (μmol/L)	2340.2	1110.5	456.8	442.5	216.6
Sodium (mmol/L)	136.5	133.0	124.6	129.9	132.9
Potassium (mmol/L)	5.7	3.0	2.61	2.8	3.6

CRRT = continuous renal replacement therapy.

The blood sodium dropped to its lowest level of 123 mmol/L after 11 hours of CRRT. The lactic acid level started to rise after 9 hours of CRRT treatment, fluctuating between 3.1 and 6.6 mmol/L. This could be attributed to the low blood osmotic pressure following hemodialysis, the relative insufficiency of intravascular volume due to hypotonic dehydration, and the accumulation of lactic acid in the microcirculation. The blood sodium and lactic acid levels gradually returned to normal after the electrolyte concentration of the replacement solution was adjusted. After 9 hours of CRRT, the blood ammonia reduced to 504.7 μmol/L. Moreover, after 24 hours of CRRT, the blood ammonia reduced to 216.6 μmol/L. Thus, CRRT was discontinued and the patient gradually regained consciousness. The tandem mass spectrometry indicated that free carnitine dramatically decreased and propionyl carnitine significantly increased. The urine organic acid test results showed that methylmalonic acid and methyl citrate levels significantly increased. In addition, 2 heterozygous mutations of the MUT gene (one from each parent) confirmed the diagnosis of methylmalonic aciduria.

MRI showed evidence of hypoglycemic encephalopathy on day 10. After 41 days in the hospital, the patient was discharged successfully with the blood ammonia fluctuating between 51.4 and 63 μmol/L, received regular follow-up care, adhered to taking l-carnitine, vitamin B12, vitamin B6, folic acid, sodium bicarbonate, arginine, and special milk powder.

Despite not experiencing any seizures, the patient’s motor development was delayed. The boy can walk with the assistance of things, has a weak ability to point to objects, can say 20 words and a few simple sentences at the age of 2.5 years old. In a recent head CT scan, no abnormalities were detected, while a percutaneous gastrostomy due to feeding difficulties was performed.

## 3. Discussion

MMA is the most common organic acidemia in China. It is caused by a deficiency in methyl malonyl coenzyme A mutase or its cofactor adenosyl cobalamin, resulting in the abnormal accumulation of metabolites like methylmalonic acid, 3-hydroxy propionic acid, and methyl citrate. The disease can inflict harm on numerous organ systems, especially the brain. Correcting the metabolic disorder is crucial during the acute phase of MMA. What’s more, the prognosis of MMA is related to the disease type and complications.

Neonatal hyperammonemia is a metabolic disorder,^[1]^ mostly observed in genetic metabolic diseases such as urea circulation disorder and organic acidemia. When the blood ammonia levels exceed 800 μmol/L for more than 24 hours, irreversible nerve damage may occur. Ammonia is a highly toxic neurotoxin. Persistent hyperammonemia can lead to an increase in both intracellular osmotic pressure and the level of extracellular potassium ions. Astrocytes metabolize ammonia into glutamine, causing cell edema. At the same time, inflammatory factors and other factors are released to damage neurons, resulting in brain edema, severe neurological consequences and even death.

The duration of coma and the level of blood ammonia can determine the prognosis.^[2]^ Therefore, it is necessary to administer emergency treatment to reduce the blood ammonia level. Current treatments for hyperammonemia include drug therapy, peritoneal dialysis, and hemodialysis (including CRRT treatment). Dialysis treatment should be carried out promptly in children with severe hyperammonemia (blood ammonia level >400–500 μmol/L) or if the blood ammonia does not significantly decrease after several hours of drug treatment.^[2–4]^

Although peritoneal dialysis is the preferred method for children’s blood purification,^[5]^ CRRT has its unique advantages for genetic metabolic diseases. Neonatal genetic metabolic diseases are typically severe, and it is crucial to remove toxic metabolites from the body quickly. CRRT often works by dialysis or filtration modes, which can continuously remove excess water and other small to medium-sized molecular metabolic substances from the body. However, peritoneal dialysis can only remove water, urea nitrogen, creatinine and other small molecular substances, but not inflammatory mediators, toxic metabolites and other medium molecular substances.^[6]^ Furthermore, CRRT has a high ammonia clearance rate and a high tolerance, assisting in reducing the high intracranial pressure caused by hyperammonemia.^[7]^ Peritoneal dialysis had limited efficacy in treating severe hyperammonemia. It was prone to complications like obstruction and catheter leakage, which could reduce the toxin clearance rate and prolong the dialysis time.

Recent researches have indicated that CRRT has become the most commonly used treatment for refractory neonatal hyperammonemia.^[1,8]^ Spinale et al^[9]^ have showed that CRRT successfully saved 2 cases of neonatal hyperammonemia caused by ornithine transaminase deficiency, achieving a high blood ammonia clearance rate. Lu Keyu et al^[1]^ have reported that CRRT was employed to treat neonatal hyperammonemia, and the blood ammonia concentration of 6 patients notably decreased. In 2020, the expert consensus on the treatment of children’s hyperammonemia indicated that CRRT was recommended for children with a blood ammonia level of >1000 μmol/L. The decision-making process should take into account the availability of dialysis equipment and/or staff, the patient’s diagnosis and overall condition, the trend in serum ammonia levels, the response to nitrogen-scavenging therapy, and the age and body weight of the patient.^[2]^ Currently, CRRT treatment is not widely adopted in neonatal intensive care units in China, and most primary hospitals lack the necessary equipment and medical staff. In the event of severe neonatal hyperammonemia, the patient should be transferred to a level III neonatal intensive care units with CRRT equipment and staff as soon as possible. Peritoneal dialysis could still be beneficial if transfer cannot be timely arranged.

The patient in this report had severe MMA with hyperammoniacal coma at admission. The blood ammonia level surged to 2000 μmol/L shortly. The patient’s blood ammonia level significantly decreased after 6 hours of CRRT treatment (the clearance rate was around 200 μmol/L/h) and gradually recovered after treatment. Currently, the use of CRRT in neonates is still restricted, and more clinical experience is required. Due to the patient’s low weight and thin blood vessels, it was not possible to insert a double-lumen hemodialysis catheter. Therefore, we used peripheral arteries and veins to insert the catheter during CRRT treatment, while the blood pump speed remained normal. Although studies have shown that citrate anticoagulant could be selected as an anticoagulant, it still requires further study. In patients with methylmalonyl coenzyme A mutase deficiency, the activity of succinate dehydrogenase decreases, which may affect the tricarboxylic acid cycle of the mitochondria.^[10]^ In this case, the increase in lactic acid during CRRT treatment might be related to the accumulation of excessive citrate in the body. It might be more appropriate to select heparin for anticoagulation in such patients.

This patient had a simple MMA mutation (0), and symptoms emerged 6 days after birth. Although the patient was saved through active and effective CRRT treatment and received regular follow-ups, he still exhibited significant overall growth retardation, which may be related to the long-term accumulation of methylmalonic acid and other metabolic products in the primary disease. During the early stage of the disease, CRRT treatment effectively removed blood ammonia and accumulated metabolites from the body, thereby laying an essential foundation for patient survival during the critical stage of the disease and creating an opportunity for late-stage treatment.

## 4. Conclusion

CRRT treatment effectively removed blood ammonia and accumulated metabolites from the body, thereby laying an essential foundation for patient survival during the critical stage of the disease and creating an opportunity for late-stage treatment.

## Author contributions

**Conceptualization:** Bingchun Lin.

**Data curation:** Bingchun Lin, Chuanzhong Yang.

**Formal analysis:** Bingchun Lin, Yichu Huang, Yanliang Yu, Xiaoyun Xiong, Chuanzhong Yang.
